# Abnormal Hypermethylation of the VDAC2 Promoter is a Potential Cause of Idiopathic Asthenospermia in Men

**DOI:** 10.1038/srep37836

**Published:** 2016-11-28

**Authors:** Aiming Xu, Yibo Hua, Jianzhong Zhang, Wei Chen, Kai Zhao, Wei Xi, Hainan Wang, Jianzheng Fang, Shifeng Su, Min Tang, Bianjiang Liu, Zengjun Wang

**Affiliations:** 1 Department of Urology, The First Affiliated Hospital of Nanjing Medical University, Nanjing 210029, China; 2 Department of Urology, Zhongshan Hospital affiliated to Fudan University, Shanghai 200032, China; 3 State Key Laboratory of Reproductive Medicine, Nanjing medical University, Nanjing 210029, China

## Abstract

This study aimed to explore the association between the methylation status of the VDAC2 gene promoter region and idiopathic asthenospermia (IAS). Twenty-five IAS patients and 27 fertile normozoospermia (NZ) were involved. GC-2spd cells were treated with different concentrations of 5-aza-2′-deoxycytidine (5-Aza-CdR) for 24 h and 48 h. qRT-PCR was conducted to reveal whether or not VDAC2 expression was regulated by methylated modification. A dual-luciferase activity detection was used to verify VDAC2 promoter activity in GC-2spd cells. Bisulphite genomic sequence was used to analyse DNA methylation of the VDAC2 promoter. The results showed that VDAC2 expression was significantly increased after treated with 5-Aza-CdR. A strong activity of the promoter (−2000 bp to +1000 bp) was detected by dual-luciferase activity detection (*P* < 0.05). The bisulphite genomic sequencing and correlation analysis showed that sperm motility was positively associated with the methylation pattern of uncomplete methylation and mild hypermethylation, and negatively related to the percentage of moderate methylation. In conclusion, high methylation of the VDAC2 promoter CpGs could be positively correlated with low sperm motility. Abnormal methylation of VDAC2 promoter may be a potential cause to idiopathic asthenospermia.

Approximately 50–80 million people worldwide are affected by infertility; of these cases, 50% are caused by male factors and most of them have asthenospermia. Varicoceles^1^, prostatitis^2^, and genital hypoplasia^3^ also result in low sperm motility. Idiopathic asthenospermia (IAS) is a disease without clear causes and is detected after regular examination and laboratory tests; IAS accounts for 30% of patients affected by male factors. According to the World Health Organization (WHO), the most defining symptom of IAS is normal sperm parameters except for low sperm motility^4^,^5^. Spermatogonium in testis and epididymis starts to mature to primary spermatocytes, secondary spermatocytes, and then sperm cells. Functional sperm contain a series of complex regulations, including gene expression, histone methylation, acetylation and promotion of sperm differentiation and maturation by microRNAs^6^. Abnormal protein expression and epigenetic modification changes may negatively affect sperm motility and morphology, thereby inducing male infertility. Abnormal regulation in spermatogenesis is a common aetiological factor of IAS.

Voltage-dependent anion channels (VDACs), also known as the mitochondrial porin protein, are pore-forming activators in the extract of *Paramecium tetraurelia* mitochondria^7^,^8^. VDAC has 3 subtypes (VDAC1, VDAC2, and VDAC3), which are approximately 70% identical and encode 3 different proteins^9^,^10^. Cheng *et al*. stated that VDAC2 is embryonically lethal and anti-apoptotic; this gene has gained increased research attention because of its roles during various physiological phenomena^11^. As the ‘gatekeeper’ of mitochondria, VDAC2 regulates and controls the transport of adenosine triphosphate/adenosine diphosphate (ATP/ADP), calcium ion (Ca^2+^) and nicotinamide adenine dinucleotide/reduced nicotinamide adenine dinucleotide (NAD/NADH)^12^,^13^,^14^. At low voltages (<10 mV), VDAC is stable in a long-lived open state and allows macromolecular solutes, such as ATP, to succinate across the planar bilayer membranes; at high positive or negative potentials (>40 mV), VDAC presents in multiple states, creating passages for select ions and proteins^15^,^16^.

VDAC2 plays an important role in spermatogenesis and male infertility^17^. It is abundant in the mitochondria outer dense fibers, which are close to the dynein light chain Tctex-type 1; it also regulates sperm motion and sperm tail structural integrity through interaction with Tctex or microtubule-associated proteins^15^,^18^,^19^. Human sperm was co-incubated with anti-VDAC antibody for 3 h, and the results indicated that the straight-line velocity (VSL), curvilinear velocity (VCL), and average path velocity (VAP) of spermatozoa were decreased via Ca^2+^ transmembrane flow inhibition^18^. Hence, abnormal VDAC2 expression is a potential cause of low sperm motility.

In our previous studies, we collected normal human adult semen samples and demonstrated, for the first time, that VDAC2 is located in human spermatozoa, specifically in sperm flagellate^20^. Furthermore, other studies have reported the expression profiles of different VDAC subtypes in mRNA in ejaculated spermatozoa from participants with normozoospermia (NZ) and patients with IAS; results found that infertility in male patients with IAS was correlated with VDAC2 expression^17^,^21^. However, just like the unclear molecular mechanism in VDAC3-lacking mice with normal parameters except for progressive motility, the underlying mechanism remains unclear^22^.

In this study, we explored the different methylation statuses of the VDAC2 gene between normal spermatozoa and asthenospermia. The relationship between sperm parameters and methylation level was determined to propose potential pathogenesis and promote clinical therapy for IAS.

## Results

### Patient Characteristics

Two separate groups, 27 participants with NZ and 25 with IAS, were recruited in the study; the mean ages of the 2 groups were 28 ± 5.56 and 28 ± 3.52 years, respectively. Sperm parameters, except for rapid sperm progressive motility and total motility (66.01 ± 8.72 and 18.75 ± 7.26, respectively; *P* < 0.01) were not significantly different between the 2 groups (Table 1).

### Gene VDAC2 was Associated with Methylation Modification Status

**Figure 1:**
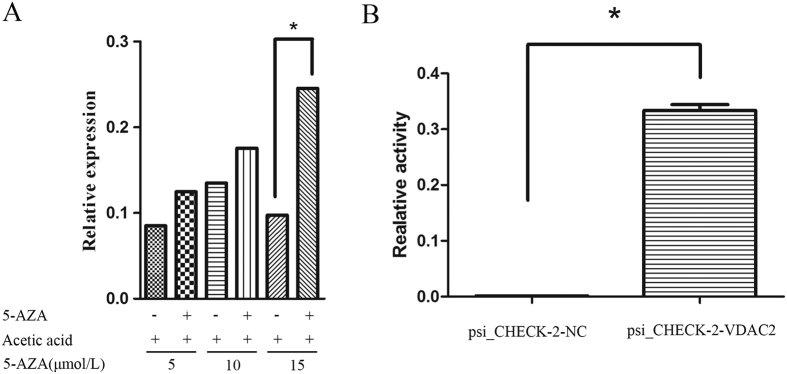
The association between VDAC2 and methylation. (**A**) Demethylation increases the expression of VDAC2. GC 2-spd cells were treated with 5, 10 and 15 μmol/L 5-Aza-CdR for 48 h. The expression of VDAC2 with 15 μmol/L dose 5-Aza-CdR was higher by threefold than that in the comparison group. (**B**) Strong promoter activity was detected by dual-luciferase reporter assay in the prognostic region −2000 bp to +1000 bp of VDAC2.

After 48 h of treatment with 5, 10, and 15 μmol/L 5-aza-2′-deoxycytidine (5-Aza-CdR) (Sigma Chemical Co., St. Louis, MO, USA), the total RNA of GC 2-spd cells was extracted, reversed, and subjected to real-time quantitive polymerase chain reaction (qRT-PCR) assessment. VDAC2 gene expression significantly increased with increasing 5-Aza-CdR dosage. Furthermore, VDAC2 expression levels increased about 3-fold after treatment with 15 μmol/L 5-Aza-CdR for 48 h compared to those in cells cultivated without 5-Aza-CdR (*P* < 0.05) (Fig. 1A). This finding indicates that demethylation enhanced VDAC2 expression.

### Strong Promoter Activity in the VDAC2 Prognostic Region −2000 bp to +1000 bp

In our previous study, strong promoter activity was detected by dual-luciferase reporter assay; hence, the prognostic region −2000 bp to +1000 bp of VDAC2 could regulate the repression of VDAC2 expression (Fig. 1B).

### CpGs (−1337 bp to −1059) may be a Potential Methylated Regulatory Region

**Figure 2:**
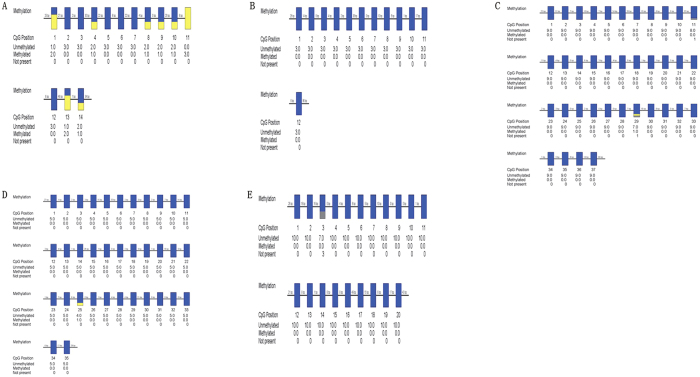
Five predicted CpG islands in VDAC2 promoter were modified by BSP. (**A**) The methylation status of CpGs from −1337 bp to −1059 bp. (**B**) The methylation status of CpGs from −163 bp to +76 bp. (**C**) The methylation status of CpGs from +123 bp to +476 bp. (**D**) The methylation status of CpGs from +630 bp to +953 bp. (**E**) The methylation status of CpGs from +933 bp to +1229 bp. CpGs: methylated shown in yellow, unmethylated shown in blue, and not present shown in grey.

We used one DNA sample to be modified with sodium bisulphite to select potential research targets from five predicted CpG islands discovered by CpGplot (Fig. 2A–E). The clone numbers were related with the DNA content, but it did not affect the credibility of our results. However, only CpGs (−1337 bp to −1059 bp) could be methylated after modification with sodium bisulfite (Fig. 2A), which might be a potential methylated regulatory region for sperm motility, Abnormal methylation of CpGs (−1337 bp to−1059 bp) may cause IAS.

### Methylation status of the VDAC2 promoter

**Figure 3:**
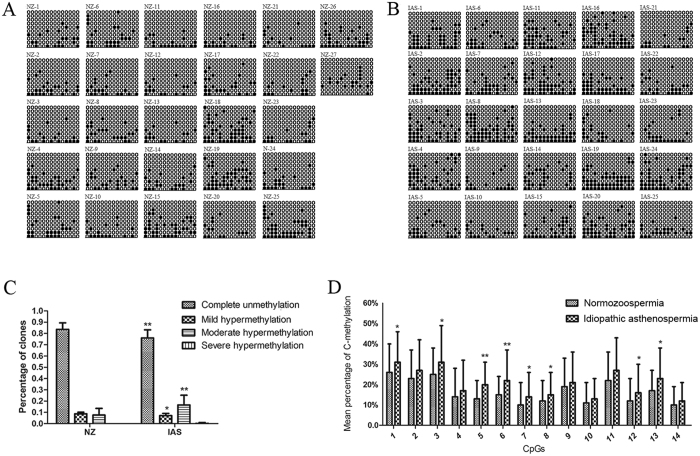
Methylation patterns of VDAC2 (14 CpGs) in human sperm. (**A**) Methylation patterns of VDAC2 promoter in normozoospermia. (**B**) Methylation patterns of the VDAC2 promoter in idiopathic asthenospermia. (**C**) Percentages of clones with four different methylation statuses of VDAC2 promoter. (**D**) Mean percentages of cytosine methylation in all 14 CpGs of VDAC2 promoter. NZ: normozoospermia; IAS: idiopathic asthenospermia; CpGs: methylated shown in black, unmethylated shown in white. Data are expressed as means ± SD (25 patients for the IAS group and 27 donors for the NZ group). Statistically significant differences from the control group (NZ) were represented with asterisks: *P < 0.05, **P < 0.01.

In NZ, the percentages of clones with complete unmethylation, mild hypermethylation, and moderate hypermethylation of VDAC2 promoters were 83.65% ± 5.51%, 8.73% ± 1.38%, and 7.62% ± 5.68%, respectively. In IAS, the corresponding percentages were 76.02% ± 6.94%, 7.14% ± 1.86%, and 16.62% ± 8.27%, respectively (*P* = 0.005, 0.02, and 0.003, respectively; Fig. 3A–C). One patient in the IAS group presented severe hypermethylation, but none did in the NZ group. Among 14 CpGs, approximately 60% (8/14) of the CpG island exhibited a higher methylation status than the other islands (Fig. 3D). Additional information on VADC2 promoter methylation in the 2 groups is shown in Fig. 3A and B and Table 2.

### Correlation Analysis Between Methylation Status and PR%

**Figure 4:**
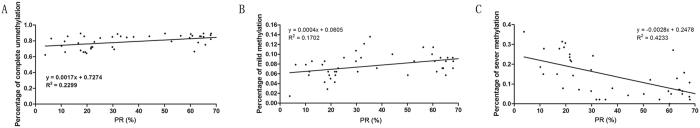
Correlation analysis between different methylation statuses of VDAC2 promoter and PR%. (**A**) Positive correlation between complete unmethylation and PR%; (**B**) Positive correlation between mild hypermethylation and PR%; (**C**) Negative correlation between moderate hypermethylation and PR%. PR%: percentage of progressive sperm ratio.

The levels of the percentage of progressive sperm ratio (PR%) were positively correlated with the percentages of complete unmethylation (r = 0.48; *P* < 0.05) (Fig. 4A) and mild hypermethylation (r = 0.41; *P* < 0.05) (Fig. 4B). Moreover, the level of progressive sperm was negatively correlated with moderate hypermethylation (r = −0.65; *P* < 0.05) (Fig. 4C, Table 3). However, methylation status was not correlated with age, semen volume, and sperm liquefaction times (Table 3).

## Discussion

In this study, we assessed genome demethylation by treating GC-2spd cells with 5-Aza-CdR. VDAC2 mRNA expression was compared between GC-2spd cells with and without 5-Aza-CdR through qRT-PCR analysis, and predicted the VDAC2 promoter region. After constructing plasmids containing the VDAC2 gene promoter, dual-luciferase activity was detected to verify the activity of the VDAC2 promoter region in GC-2spd cells. The extract of 52 sperm genomic DNA samples was subjected to bisulfite sequencing through PCR to analyze the DNA methylation of the VDAC2 promoter and determine the causes of down-regulated VDAC2 mRNA expression in IAS.

The VDAC2 protein is involved in many physiological functions, such as anti-apoptosis^11^, metabolite transport, spermatogenesis^15^, and oogenesis^23^. In the present study, the high expression of VDAC2 mRNA in the demethylation group indicated that VDAC2 expression was associated with the methylation status of the promoter regions; however, whether hypermethylation leads to down-regulation of the VDAC2 protein remains unknown. The VDAC2 protein contains 9 cysteines, removal or modification of which can alter channel function in human^24^,^25^. For example, specific modification of zinc finger cysteines with methyltransferase activity could abolish ubiquitin-chain binding of the Npl4 zinc finger (NZF) domains in TAB2/3, thereby disrupting host NF-kB signaling^26^. During methylation, where nucleophilic attack of cytosine C5 on the S-adenosyl-L-methionine methyl group occurs, a covalent bond between Cys81-S^−^ and cytosine C6 was rapidly formed; this bond can form and break reversibly^27^,^28^. Based on the conserved cysteine components at corresponding regions in VDAC2, we speculated that an analogous change in the thiol status upon oxidative stress can lead to the closure of VDAC2 channels^29^. Therefore, methylation in the CpGs of the VDAC2 promoter region, which results in down-regulation of the VDAC2 protein or dysfunction of cysteine components, must be further studied.

Vector construction is an important tool for molecular biological processes, including renovation of known carrier polyclonal sites and functional components, such as promoters, enhancers, and biomarkers. This study was the first to construct the vector of the VDAC2 gene promoter and lays a solid foundation for further studies on the transcriptional activity of the promoter region and nuclear promoter regions and the transcriptional regulatory mechanism of VDAC2. Considering the difficulty in acquiring human spermatocytes, we transfected the vector plasmid into the mouse spermatogenic GC-2spd cells. Strong activity of the promoter (−2000 bp to +1000 bp) was detected by dual-luciferase activity analysis (*P* < 0.05). Based on previous studies, we suggest that abnormal methylation of the VDAC2 promoter region causes either a decrease in the expression of VDAC2 mRNA or a functional defect, leading to changes in sperm motility and male infertility.

In this study, our results showed that clones with <80% methylation (i.e., complete unmethylation, mild hypermethylation, and moderate hypermethylation) of VDAC2 promoter were found in both two groups, whereas clones with >80% methylation (i.e., sever hypermethylation) occurred only in the IAS group. We further compared the correlation of different methylation categories of VDAC2 promoter region and sperm motility. In complete unmethylation or mild hypermethylation, sperm motility improved with increasing degree of methylation, especially in the situation of complete unmethylation. The acceleration rate of progressive motility decreased in mild methylation sperm compared with unmethylation sperm (as shown in Fig. 4A and B, with a slope of 0.0004 versus 0.0017). Meanwhile, the slope for severe methylation sperm was −0.0028, which was less than the slope for mild methylation sperm (as shown in Fig. 4B and C, with a slope of −0.0028 versus 0.0004). The rate of acceleration of sperm progressive motility decreased with the degree of hypermethylation. Based on previous studies, hypermethylation of the CpG island (−1337 bp to −1059 bp) may decrease the expression of VDAC2 mRNA and exhibit untoward effects on subsequent translation, which may inactive the VDAC2 protein, reduce of ATP/ADP, Ca^2+^, NAD/NADH and other small molecules across the mitochondrial outer membrane, weaken sperm flagellae, and ultimately result in IAS. Our results suggested that abnormal hypermethylation of the CpG island of the VDAC2 promoter resulted in sperm with slow speed.

In conclusion, methylation regulates body growth and development, and is essential for normal metabolic and organ functions under any circumstances. VDAC2 is important to sperm motility, which depends on the methylation status of the CpG island of the its promoter region. Abnormal hypermethylation of VDAC2 promoter region, especially in CpG island (−1337 bp to −1059 bp), might down-regulate the protein expression or led to abnormal protein functions, ultimately result in IAS. However, all conclusions are based on the limited sample size employed in the study. A large sample size is therefore needed to confirm our findings. Furthermore, the molecular mechanism of the CpG island (−1337 bp to −1059 bp) regulating sperm motility must be discussed.

## Materials and Methods

### Patient Recruitment and Semen Classification

Subjects were recruited from the andrology clinic of the First Affiliated Hospital of Nanjing Medical University in Nanjing, China. All subjects gave signed informed consent before participating in the study. Prior to sample collection, this study was approved by the ethics committee of Nanjing Medical University (China). The semen analyses were conducted by the chief laboratorian Ting Wu using a spermatozoa analyzer (CASA, WLJY 9000, Wei li New Century Science and Tech Dev, Beijing, China). Routine semen assessments were conducted according to the fourth edition of the WHO Laboratory Manual for the Examination and Treatment of Human Semen. Twenty-seven NZ semen samples were used as the control group and met the following requirements: liquefaction time (min) <60; volume (ml) ≥2; sperm concentration (×10^6^/ml) ≥20; motility (%) ≥70; PR (%) ≥50; leukocytes (×10^6^/ml) <1; and pH 7.2–7.8. The 25 IAS participants met the same standards except for PR (%) <50 or rapid motility (%) <25. These patients failed to impregnate their wives for at least 2 years. All semen samples were collected by masturbation after 3–5 days of abstinence. Routine physical examination showed that the participants were well-developed men. No acute or chronic inflammation, anti-sperm antibodies, or varicoceles were found. Serum testosterone, luteinizing hormone, and follicle-stimulating hormone levels were within normal ranges.

### Cell Culture and 5-Aza-CdR Treatment

GC-2spd cells from mouse spermatocytes were used to study spermatogenesis and were donated by the State Key Laboratory of Reproductive Medicine. GC-2spd cells were grown in Dulbecco’s modified eagle medium (DMEM) (Gibco, Grand Island, USA), supplemented with 10% fetal bovine serum and 1% penicillin–streptomycin at 37 °C in 5% CO_2._ Upon reaching 80% to 90% confluence, total cells were washed twice with phosphate buffer (PBS), trypsinized with 0.25% trypsin (Gibco, Grand Island, USA), and seeded in a 6-well plate.

5-Aza-CdR was dissolved in 50 mg/ml 50% acetic acid, and the solutions were stored in ice until use according to the manufacturer’s instructions. 5-Aza-CdR treatment was optimized to establish a working concentration range of 5 μmol/L to 15 μmol/L. Cells were exposed to 5-Aza-CdR for 24 and 48 h to allow drug incorporation into DNA. Control samples were added with the same volume of solvent to determine whether VDAC2 was regulated by methylation.

### Total RNA Extraction and qRT-PCR

After 5-Aza-CdR treatment for 24 and 48 h, GC-2spd cells were washed twice with PBS and lysed by Trizol reagent (Invitrogen, Carlsbad, USA) according to the manufacturer’s instructions. Briefly, 1 μg of RNA was reverse transfected, and 2 μl of cDNA was amplified as follows: 95 °C for 30s, 40 cycles at 95 °C for 5s, and 60 °C for 1 min. The primers for VDAC2 and β-actin are displayed in Table 4. Three replicates of each reaction were performed, and cycle threshold values were averaged. The reactions were performed and analyzed via an ABI 7900 system (Applied Biosystems, Carlsbad, USA).

### Plasmids and Promoter Reporter Constructs

**Figure 5:**
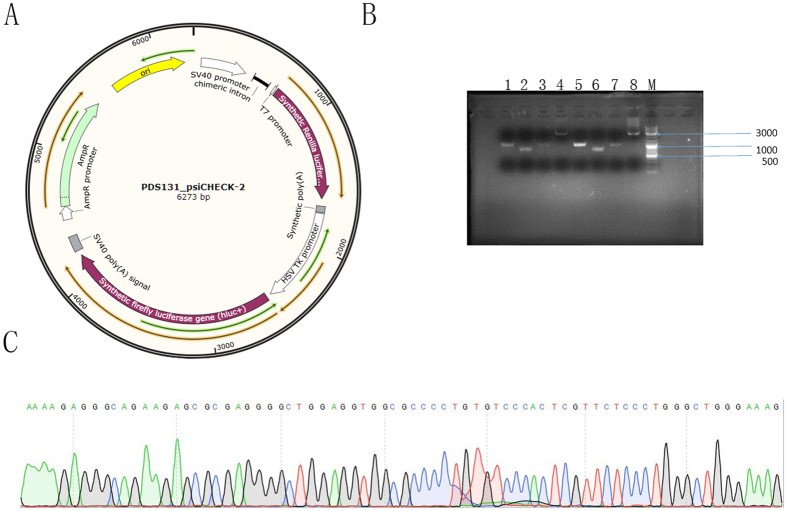
Identification of the human VDAC2 gene promoter. (**A**) psi_CHECK-2. (**B**) M: 3000 bp DNA Marker, 1–4 represent the brands of psi_CHECK-2-VDAC2 and replied with 5–8. The well of 1, 2, 3, 5, 6 and 7 were the fractional products of the VDAC2 promoter region; the well of 4 and 8 were the integrated products. (**C**) Partial sequence of the product of the recombined VDAC2 promoter region.

A psi_CHECK-2 vector was purchased from Novobio, Shanghai (Fig. 5A). The entire sequence of the predicted VDAC2 promoter region (−2000 bp to +1000 bp) was correctly synthesized (Fig. 5B) and inserted into the psi_CHECK-2 vector between *Kpn* I and *Nhe* I restriction enzyme cutting sites; the vector was named psi_CHECK-2-VDAC2. The product was transferred into competent DH5α cells, which were cultured overnight. Bacterial colonies were randomly picked for sequencing. Correct sequences were ensured after amplification with an automatic sequencer (Fig. 5C). A GenElute Plasmid Miniprep Kit was used for extraction and collection of recombinant plasmid according to the manufacturer’s instruction. The plasmid was stored at −20 °C until use.

### Dual-luciferase Reporter Assay

Luciferase assays were performed according to the manufacturer’s protocol. GC-2spd cells were seeded into a 6-well plate at a density of 5 × 10^5^ per well. Upon reaching 50–60% confluence, GC-2spd cells were transfected with psi_CHECK-2-VDAC2 and psi_CHECK-2-NC, which was used as negative control through Lipofectamine 2000 (Invitrogen, Carlsbad, USA). At 48 h after transfection, the cells were lysed and luciferase activity was examined via Dual-Luciferase Reporter Assay System (Promega E1910, Wisconsin, USA). Data were recorded on a luminometer (Tecan infinite M200 pro, Austria) and normalized by dividing firefly luciferase activity with that of Renilla luciferase. The data were then analyzed and graphed using Excel (Microsoft).

### DNA Extraction and Bisulfite Genomic Sequencing of the VDAC2 Promoter CpGs

DNA was extracted from all semen samples using a genomic DNA isolation kit (Generay, Shanghai, China). Isolated genomic DNA was modified with the sodium bisulfite method using an EpiTect Fast DNA Bisulfite Kit according to the manufacturer’s protocol (Qiagen 59824, Germany). The PCR primers for the predicted promoter region after bisulfite conversion were F: TTTAATATTTTG GTTAATATGGTGAAATTT and R: AAAACTCCCAAAACAATCATCTATC. The reaction for mRNA detection was performed according to the following conditions: 95 °C for 4 min, 40 cycles at 94 °C for 30s, 50 °C for 30s, and 72 °C for 40s. The reactions were performed and analyzed via an ABI 7900 system. For sequence analysis after methylation, products were cloned into the pTG19-T vector (Generay, Shanghai, China) and 10 individual clones were sequenced.

### Statistical Analysis

Methylation status was categorized according to methylation degree into 4 types: complete unmethylation (no methylated CpGs), mild hypermethylation (0–20% methylated CpGs), moderate hypermethylation (20–80% methylated CpGs) and severe hypermethylation (80–100% methylated CpGs)^30^. Student’s *t*-test was performed to compare methylation status between the 2 groups. Correlation analysis was applied to evaluate the relationship between different methylation types and sperm parameters. Results of bisulfite genomic sequencing were analyzed via BiQ_Analyzer. All statistical data were presented as mean ± SD and analyzed using SPSS 20.0. *P* < 0.05 was considered statistically significant, and *P* < 0.01 was considered extremely significant.

## Additional Information

**How to cite this article**: Xu, A. *et al*. Abnormal Hypermethylation of the VDAC2 Promoter is a Potential Cause of Idiopathic Asthenospermia in Men. *Sci. Rep.* **6**, 37836; doi: 10.1038/srep37836 (2016).

**Publisher's note:** Springer Nature remains neutral with regard to jurisdictional claims in published maps and institutional affiliations.

## Figures and Tables

**Table 1:** Sperm characteristics of the analysed patient cohort.

Type	Number	Age (year)	Motility (%)*	Concentration (×10^6^)	Volume (ml)
NZ	27	28 ± 5.56	66.01 ± 8.72	123.85 ± 48	3.16 ± 1.03
IAS	25	28 ± 3.52	18.75 ± 7.26	120.08 ± 38.64	3.65 ± 1.88

Data were presented as means ± SD, groups with different superscripts differ significant (*P* < 0.05 by ANOVA).

NZ: normozoospermia; IAS: idiopathic asthenospermia; *P < 0.05.

**Table 2:** Methylation status of VDAC2 in human sperm in NZ and IAS group.

Groups	Status of methylation	CpG1 a%(n/N)	CpG2a%(n/N)	CpG3a%(n/N)	CpG4a%(n/N)	CpG5a%(n/N)	CpG6a%(n/N)	CpG7a%(n/N)
NZ	Complete unmethylation (0%)**	74.07%(200/270)	76.67%(207/270)	75.19%(203/270)	86.30%(233/270)	86.67%(234/270)	84.81%(229/270)	90.37%(244/270)
	Mild methylation (0–20%)*	7.41%(20/270)	8.15%(22/270)	9.26%(25/270)	6.67%(18/270)	11.85%(32/270)	10.74%(29/270)	7.04%(19/270)
	Moderate methylation (20–80%)**	18.52%(50/270)	15.19%(41/270)	15.56%(42/270)	7.04%(19/270)	1.48%(4/270)	4.44%(12/270)	2.59%(7/270)
	Severe methylation (80–100%)	0	0	0	0	0	0	0
	Total (N)	270	270	270	270	270	270	270
**Groups**	**Status of methylation**	**CpG1a%(n/N)**	**CpG2a%(n/N)**	**CpG3a%(n/N)**	**CpG4a%(n/N)**	**CpG5a%(n/N)**	**CpG6a%(n/N)**	**CpG7a%(n/N)**
IAS	Complete unmethylation (0%)	64.00%(160/250)	69.20%%(173/250)	63.60%(159/250)	80.80%(202/250)	75.60%(189/250)	72.80%(182/250)	80.80%(202/250)
	Mild methylation (0–20%)	3.60%(9/250)	6.40%(16/250)	4.00%(10/250)	6.40%(16/250)	6.00%(15/250)	4.40%(11/250)	8.80%(22/250)
	Moderate methylation (20–80%)	32.40%(81/250)	24.40%(61/250)	29.20%(73/250)	12.80%(32/250)	18.40%(46/250)	22.80%(57/250)	10.40%(26/250)
	Severe methylation (80–100%)	0	0	3.20% (8/250)	0	0	0	0
	Total (N)	250	250	250	250	250	250	250
**Groups**	**Status of methylation**	**CpG8a%(n/N)**	**CpG9a%(n/N)**	**CpG10a%(n/N)**	**CpG11a%(n/N)**	**CpG12a%(n/N)**	**CpG13a%(n/N)**	**CpG14a%(n/N)**
NZ	Complete unmethylation (0%)**	88.15%(238/270)	80.74%(218/270)	89.26%(241/270)	77.78%(210/270)	88.15%(238/270)	83.33 (225/270)	89.63%(242/270)
	Mild methylation (0–20%)*	8.15%(22/270)	8.15%(22/270)	8.15%(22/270)	9.26%(25/270)	8.15%(22/270)	10.00%(27/270)	9.26%(25/270)
	Moderate methylation (20–80%)**	3.70%(10/270)	11.11%(30/270)	2.59%(7/270)	12.96%(35/270)	3.70%(10/270)	6.67%(18/270)	1.11%(3/270)
	Severe methylation (80–100%)	0	0	0	0		0	0
	Total (N)	270	270	270	270	270	270	270
**Groups**	**Status of methylation**	**CpG1a%(n/N)**	**CpG2a%(n/N)**	**CpG3a%(n/N)**	**CpG4a%(n/N)**	**CpG5a%(n/N)**	**CpG6a%(n/N)**	**CpG7a%(n/N)**
IAS	Complete unmethylation (0%)	81.60%(204/250)	76.40%(191/250)	85.20%(213/250)	69.60%(174/250)	80.80%(202/250)	72.80%(182/250)	86.40%(216/250)
	Mild methylation (0–20%)	8.00%(20/250)	8.80%(22/250)	10.40%(26/250)	5.60%(14/250)	8.00%(20/250)	6.40%(16/250)	9.60%(24/250)
	Moderate methylation (20–80%)	10.40%(26/250)	14.80%(37/250)	4.40%(11/250)	24.80%(62/250)	11.20%(28/250)	20.80%(52/250)	4.00%(10/250)
	Severe methylation (80–100%)	0	0	0	0	0	0	0
	Total (N)	250	250	250	250	250	250	250

CpG:—C—phosphate—G—; NZ: normozoospermia; IAS: idiopathic asthenospermia; Statistically significant differences from the control group (NZ) were represented with asterisks: *P < 0.05, **P < 0.01.

**Table 3:** Correlation between different methylation status of VDAC2 promoter and PR%.

Variables	Correlation coefficient (r)		
	Completely unmethylation (0%)	Mild methylation (0–20%)	Moderate methylation (20–80%)
Age	−0.192	−0.163	0.201
PR%	0.48^**^	0.41^**^	−0.65^**^
Density	0.177	−0.024	−0.141
Volume	0.103	0.085	−0.107

PR%: percentage of progressive sperm ratio; *P < 0.05; **P < 0.01.

**Table 4:** PCR primers used in this study.

Name	Site	Primer sequence (5′–3′)	Annealing temp. (°C)	Product size (bp)	CpGs (n)
*CpG island-1*	*−1337~−1059*	F: TTTAATATTTTGGTTAATATGGGTGAAATTT	70.4	279	14
		R: AAAACTCCCAAAACACTCATCTATC			
*CpG island-2*	*−163~* +*76*	F: TTTATAATTTAGAGGAGGTTGGGTT	70.1	250	12
		R: CTACCCTCTTTTCCCACCTTAATATA			
*CpG island-3*	+*123~* +*476*	F: TTTTTGGGTTGGGAAAGAGA	70.4	354	37
		R: AACTACTAAACCTTAAATCAAACAAC			
*CpG island-4*	+*630~* +*953*	F: TTTAAGGTTTTGTTTGGGAGAGGATAG	69.8	324	35
		R: ACTAAAATCAAAAAACCAAAAAAAA			
*CpG island-5*	+*933~* +*1229*	F: TTTTGTTTTTTTGATTTTAGTTTAT	70.0	297	20
		R: AACCTTAATTCCCCATTTAAAATCC			
VDAC2	*—*	F: AGAGAAGCGGTCCCTCAGAA	68.9	100	*—*
		R: AAACAAAGCCCTGCATAGGGT			
β-actin	*—*	F: TCACCCACACTGTGCCCATCTACGA	72.3	295	*—*
		R: CAGCGGAACCGCTCATTGCCAATGG			
